# Clinical indices and anthropometric measures for magnetic resonance imaging-defined visceral adiposity across the hypercortisolism spectrum

**DOI:** 10.3389/fendo.2026.1959212

**Published:** 2026-09-09

**Authors:** Aslihan Pekmezci, Mehmet Ali Arikan, Idris Kuzu, Merve Korkmaz Yilmaz, Ceyda Ceren Arikan, Sebnem Burhan, Rukiye Dilara Uzman Tekin, Didem Acarer Bugun, Aytul Hande Yardimci, Mutlu Niyazoglu, Esra Hatipoglu

**Affiliations:** 1 Department of Endocrinology and Metabolism, University of Health Sciences, Basaksehir Cam and Sakura City Hospital, Istanbul, Türkiye; 2 Department of Radiology, University of Health Sciences, Basaksehir Cam and Sakura City Hospital, Istanbul, Türkiye; 3 Department of Endocrinology and Metabolism, Medicana Atakoy Hospital, Istanbul, Türkiye; 4 University of Health Sciences, Pituitary Disorders Research Center, Istanbul, Türkiye

**Keywords:** Cushing’s disease (CD), hepatic steatosis index (HSI), magnetic resonance imaging (MRI), mild autonomous cortisol secretion (MACS), visceral adipose tissue (VAT), visceral adiposity index (VAI)

## Abstract

**Objective:**

Conventional anthropometric measures may inadequately capture metabolically relevant fat redistribution in hypercortisolism. We evaluated the discriminative performance of clinical indices (Hepatic Steatosis Index [HSI], Visceral Adiposity Index [VAI], Metabolic Score for Visceral Fat [METS-VF]) and anthropometric measures (body mass index [BMI], waist circumference) for MRI-defined visceral adiposity across the hypercortisolism spectrum.

**Design and methods:**

In this cross-sectional study, 65 participants were included: 13 with active Cushing’s disease (CD), 12 with CD in remission, 30 with mild autonomous cortisol secretion (MACS), and 10 healthy controls. Abdominal MRI with proton density fat fraction separately quantified visceral (VAT), hepatic, and pancreatic fat. Index performance for visceral adiposity (VAT >130 cm²) was assessed among participants with hypercortisolism (n=55).

**Results:**

VAT differed across groups (p=0.02), highest in MACS (230.8 [164.9–292.4] cm²). VAT was associated with HOMA-IR (r=0.5, p=0.009) and HbA1c (r=0.4, p=0.001) but not with circulating cortisol. Hepatic and pancreatic fat did not differ across groups; hepatic fat correlated with HOMA-IR and pancreatic fat with HbA1c, but neither with cortisol. HSI was the only index reaching significance for visceral adiposity (AUC 0.82, 95% CI: 0.68–0.96, p=0.002), at a cohort-derived cut-off of 39.25 (sensitivity 87%, positive predictive value 93%); METS-VF, BMI, and waist circumference did not (all p≥0.1). Among participants without obesity (BMI <30 kg/m², n=31), HSI classification remained associated with visceral adiposity (p=0.001).

**Conclusion:**

In endogenous hypercortisolism, ectopic fat distribution was more closely associated with metabolic burden rather than cortisol levels. HSI may have potential as an exploratory screening adjunct for occult visceral adiposity, particularly in patients without obesity, pending external validation.

## Introduction

Endogenous hypercortisolism is associated with a characteristic cardiometabolic phenotype marked by central adiposity, impaired glucose metabolism, dyslipidemia, hepatic steatosis, and an increased cardiovascular risk (1, 2). This burden is linked not to overall obesity but to the expansion of visceral adipose tissue (VAT) and ectopic fat deposition in the liver and pancreas, where excess lipid accumulation drives insulin resistance, metabolic dysfunction-associated steatotic liver disease (MASLD), and impaired beta-cell function independently of total body fat (2–6).

Chronic glucocorticoid excess promotes preferential visceral fat accumulation through depot-specific effects on adipocyte differentiation, lipolysis, and lipid trafficking (2, 7, 8). This redistribution, disproportionately expanding visceral fat at the expense of peripheral subcutaneous stores, constitutes the core metabolic phenotype of both overt Cushing syndrome (CS) and mild autonomous cortisol secretion (MACS) and underlies much of the associated cardiometabolic risk (5, 7, 9). Whether it is driven primarily by the magnitude of cortisol exposure or by its downstream metabolic consequences, including insulin resistance, dyslipidemia, and impaired glucose homeostasis, remains an open question with direct implications for risk stratification (9–11).

Magnetic resonance imaging–derived proton density fat fraction (MRI-PDFF) enables direct, quantitative assessment of ectopic fat across multiple compartments and represents the reference standard for non-invasive fat quantification (12, 13). As advanced imaging is not routinely feasible in clinical practice, identifying which clinical tools best approximate imaging-defined adiposity in hypercortisolism carries practical relevance.

Body mass index (BMI) and waist circumference are the most widely used adiposity measures and are embedded in metabolic syndrome definitions and cardiovascular risk frameworks (14, 15). However, both reflect overall body habitus rather than compartmental distribution and provide limited information on ectopic lipid deposition in metabolically active organs (15, 16). Because cortisol-driven redistribution toward visceral and ectopic depots may occur even when external body size remains modest, anthropometric measures may fail to capture metabolically relevant adiposity in this population, in which their performance has been little studied (14, 15).

Several composite clinical indices have been proposed as indirect surrogates of visceral fat: the Visceral Adiposity Index (VAI), integrating anthropometric and lipid parameters (17); the Metabolic Score for Visceral Fat (METS-VF), incorporating insulin resistance–related components (18); and the Hepatic Steatosis Index (HSI), combining liver enzyme ratios, BMI, sex, and diabetes status, originally developed for hepatic steatosis screening but with potential metabolic utility beyond the liver (19, 20). Each was developed in general-population cohorts where metabolic derangements largely parallel fat accumulation, an assumption that may not hold in glucocorticoid excess, where glucocorticoids can induce insulin resistance through receptor-mediated pathways that are not fully captured by visceral fat volume (3, 7, 21).

In this study, we addressed two complementary questions. First, we evaluated how well HSI, VAI, METS-VF, BMI, and waist circumference discriminate MRI-PDFF–defined visceral fat accumulation in patients with hypercortisolism. Second, we examined which hormonal and metabolic parameters are associated with compartment-specific fat deposition across the visceral, hepatic, and pancreatic compartments.

## Materials and methods

### Study design and population

This cross-sectional study was conducted between November 2025 and March 2026 at the Department of Endocrinology and Metabolism, Basaksehir Cam and Sakura City Hospital, and comprised 65 participants: 25 patients with Cushing’s disease (CD) (13 active and 12 in remission), 30 with mild autonomous cortisol secretion (MACS), and 10 healthy controls (HC). The study followed the Declaration of Helsinki (1975, revised 2013), was approved by the Ethics Committee of Basaksehir Cam and Sakura City Hospital (approval number: 403/11.2025; date: November 5, 2025), and written informed consent was obtained from all participants.

Cushing’s disease was defined as endogenous hypercortisolism secondary to an adrenocorticotropic hormone (ACTH)–secreting pituitary adenoma, confirmed by standard biochemical testing and pituitary imaging (22). Active disease and remission were classified using guideline-recommended biochemical criteria, including normalization of late-night salivary cortisol (LNSC), 24-hour urinary free cortisol (UFC), and adequate suppression on dexamethasone suppression testing (DST; serum cortisol <1.8 µg/dL) (23). MACS was defined by biochemical evidence of cortisol autonomy, established as a post-1 mg overnight DST serum cortisol >1.8 µg/dL in the absence of overt Cushingoid features, in accordance with the 2023 European Society of Endocrinology guideline (24). MRI and biochemical assessments were not necessarily performed on the same day but were obtained within the same clinical evaluation period, with a median interval of 28 [14–49] days and a maximum interval of three months. Disease status and treatment remained unchanged between the two assessments.

Control participants were drawn from a previously conducted institutional thesis study (ethics approval number: 104/02.2024; date: February 14, 2024) by sex-matched randomization. Because the available control pool was limited to this pre-existing cohort, matching was restricted to sex and could not be extended to age or BMI. Control participants were required to have no history of diabetes mellitus, hypertension, or dyslipidemia; no known chronic inflammatory, endocrine, or hepatic disorders; no prior glucocorticoid exposure or use of medications known to affect adipose tissue distribution or metabolic status; and normal baseline hormonal evaluation. Exclusion criteria for all participants included chronic alcohol consumption (>20 g/day women, >30 g/day men) assessed Alcohol Use Disorders Identification Test-Concise (AUDIT-C) (25), active malignancy, viral hepatitis, other hepatic diseases of established etiology, and insufficient image quality precluding reliable PDFF quantification.

### MRI protocol and image analysis

Abdominal MRI was performed on an Ingenia 1.5T system (Philips Medical Systems, The Netherlands) with a 16-channel abdominal coil. Proton density fat fraction was quantified using a chemical shift–based mDixon-Quant sequence with 3D breath-hold acquisition (detailed parameters in **Supplementary Methods**), analyzed on the scanner’s dedicated post-processing workstation.

Hepatic PDFF was measured via circular regions of interest (ROIs) on multiple slices of the right hepatic lobe, carefully avoiding major vessels, bile ducts, focal lesions, liver margins, and imaging artifacts; mean values were used. Hepatic steatosis was defined as PDFF ≥5% (26, 27). The pancreas was assessed in three anatomically defined segments (head, body, tail; segment boundaries detailed in **Supplementary Methods**), with freehand ROIs traced at the largest cross-sectional area of each segment, excluding surrounding fat, vascular structures, and the main pancreatic duct. As volumetric segmentation was not performed, weighted mean pancreatic PDFF was calculated using published estimates of relative segment volumes (head 17.5%, body 47.5%, tail 32.5%) (28, 29); pancreatic steatosis was defined as weighted mean PDFF ≥7.9% (30).

Visceral adipose tissue (VAT) and subcutaneous adipose tissue (SAT) areas were quantified on axial T2-weighted images at the L2–L3 intervertebral level using freehand ROIs and expressed in cm²; visceral adiposity was defined as VAT >130 cm², a threshold associated with increased cardiometabolic risk in population-based studies (31–34).

**Figure 1:**
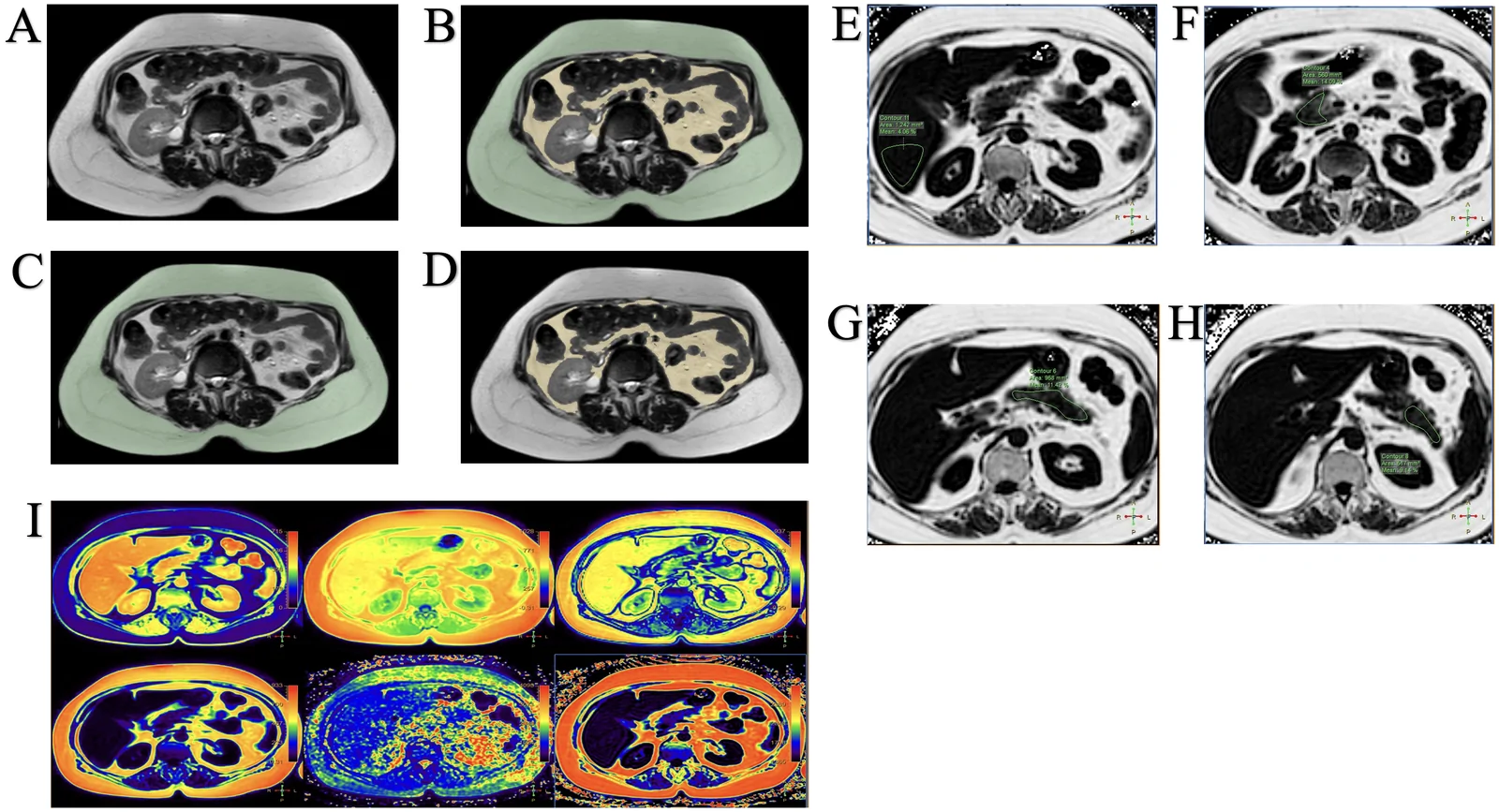
MRI-based assessment of abdominal adipose tissue distribution and ectopic fat deposition: representative examples of visceral and subcutaneous adipose tissue segmentation and PDFF mapping. **(A–D)** Representative axial images illustrating segmentation of abdominal adipose tissue compartments: **(A)** original image, **(B)** combined segmentation of visceral adipose tissue (VAT, beige) and subcutaneous adipose tissue (SAT, green), **(C)** isolated SAT, and **(D)** isolated VAT. **(E–H)** Placement of regions of interest (ROIs) for fat quantification in the liver **(E)** and pancreas, including the head **(F)**, body **(G)**, and tail **(H)**. **(I)** Representative proton density fat fraction (PDFF) map illustrating abdominal fat distribution. PDFF values were derived from multi-echo MRI sequences and expressed as percentages.

All MRI measurements were independently performed by two radiologists with experience in abdominal imaging, each blinded to clinical and biochemical data. Interobserver agreement was excellent across all compartments (liver ICC = 0.988, pancreatic head ICC = 0.988, pancreatic body ICC = 0.984, pancreatic tail ICC = 0.978); Bland–Altman analysis demonstrated minimal bias with narrow limits of agreement across all regions (**Supplementary Figure 1**; **Supplementary Methods**). The mean of the two readers’ measurements was used for all subsequent analyses. Representative examples of adipose tissue segmentation, ROI placement, and PDFF mapping are illustrated in **Figure 1**.

### Clinical, anthropometric, and biochemical assessment

Body mass index was calculated as weight (kg) divided by height squared (m²). Waist circumference was measured at the level of the umbilicus in the standing position by a single trained investigator, in accordance with WHO recommendations (35). Elevated waist circumference was defined as ≥83 cm in women and ≥93 cm in men, per Turkish population-specific thresholds (36, 37). Blood pressure was measured in the seated position after five minutes of rest, and the mean of two consecutive readings was recorded. Diabetes mellitus, hypertension, and dyslipidemia were defined according to ADA, ESC, and NCEP-ATP III criteria, respectively, or current use of disease-specific medications (38–40).

All biochemical and hormonal analyses were performed in the central laboratory of our institution. Blood samples were collected in the early morning after an overnight fast of at least eight hours. Biochemical parameters included fasting plasma glucose, fasting insulin, HbA1c, triglycerides, high-density lipoprotein cholesterol (HDL-C), low-density lipoprotein cholesterol (LDL-C), alanine aminotransferase (ALT), and aspartate aminotransferase (AST). Insulin resistance was estimated using the homeostasis model assessment of insulin resistance (HOMA-IR): [fasting insulin (μU/mL) × fasting glucose (mg/dL)]/405 (41). The Hepatic Steatosis Index was calculated as: HSI = 8 × (ALT/AST ratio) + BMI (+2 if female; +2 if diabetes mellitus), with values <30 indicating low risk and values >36 indicating high risk for hepatic steatosis (19). The Visceral Adiposity Index was calculated sex-specifically as follows: in males, VAI = [waist circumference (cm)/(39.68 + 1.88 × BMI)] × [triglycerides (mmol/L)/1.03] × [1.31/HDL-C (mmol/L)]; in females, VAI = [waist circumference (cm)/(36.58 + 1.89 × BMI)] × [triglycerides (mmol/L)/0.81] × [1.52/HDL-C (mmol/L)] (17). The Metabolic Score for Visceral Fat was calculated according to the original Bello-Chavolla formulation. First, METS-IR was calculated as: METS-IR = ln{[(2 × fasting glucose) + triglycerides] × BMI}/ln(HDL-C), with fasting glucose, triglycerides, and HDL-C expressed in mg/dL. METS-VF was then calculated as: METS-VF = 4.466 + 0.011 × [ln(METS-IR)]³ + 3.239 × [ln(waist-to-height ratio)]³ + 0.319 × sex + 0.594 × ln(age), where waist-to-height ratio = waist circumference/height and sex was coded as 1 for men and 0 for women (18).

Hormonal evaluation included morning serum cortisol, plasma adrenocorticotropic hormone (ACTH), dehydroepiandrosterone sulfate (DHEAS), late-night salivary cortisol (upper limit of normal <0.4 µg/dL), and 24-hour urinary free cortisol (UFC; upper limit of normal <45 µg/24h). The 1-mg overnight and 2-day low-dose (2 mg/day) DST were performed per standard protocols (22); salivary cortisol and UFC were each measured twice, and the mean value was used for analysis. Assay platforms and methods for all biochemical and hormonal parameters are detailed in **Supplementary Methods**.

### Statistical analysis

All statistical analyses were performed using IBM SPSS Statistics for Windows, version 26.0 (IBM Corp., Armonk, NY, USA). Figures were generated using GraphPad Prism version 11.0 (GraphPad Software, San Diego, CA, USA) and Python 3.x (matplotlib library). Normality was assessed using the Shapiro-Wilk test. Continuous variables are presented as median (interquartile range) and categorical variables as frequency and percentage. Group differences were evaluated using the Kruskal-Wallis test with pairwise Mann-Whitney U *post-hoc* comparisons; given the number of pairwise comparisons across four groups, Bonferroni correction was applied throughout as the more conservative approach, and Bonferroni-adjusted p-values are reported. Categorical variables were compared using chi-square or Fisher’s exact test, as appropriate. Associations between fat compartment variables and hormonal and metabolic parameters within the hypercortisolism cohort were assessed using Spearman rank correlation. Correlation analyses were exploratory, and no formal adjustment for multiple comparisons was applied. Multiple linear regression analysis was performed to identify independent predictors of VAT area, with BMI, diabetes status, sex, ALT, and AST entered as covariates; age was not included, as the model was designed to evaluate the independent contributions of HSI-constituent variables rather than to serve as a general predictive model for VAT.

Receiver operating characteristic (ROC) curve analyses were conducted within participants with hypercortisolism (n=55) to evaluate the discriminative performance of HSI, METS-VF, BMI, and waist circumference for VAT >130 cm²; VAI was not carried forward to ROC analysis, as it did not demonstrate significant group-level differences. Optimal cut-off values were identified using the Youden index. Within the BMI <30 kg/m² subgroup, the discriminative performance of HSI for visceral adiposity was evaluated using Fisher’s exact test, comparing HSI risk classification against MRI-defined VAT status. All tests were two-sided, and a p-value of <0.05 was considered statistically significant. *Post-hoc* statistical power for the primary ROC analysis was estimated for the observed HSI AUC against a null value of 0.5, using the standard error of the AUC derived by the method of Hanley and McNeil (42), for a two-sided test at α = 0.05. Internal validation of the HSI ROC analysis was performed using bootstrap optimism correction (2000 resamples) and leave-one-out cross-validation of the Youden-derived cut-off. Pairwise differences between the AUC of HSI and those of the competing indices were assessed using the DeLong test for correlated ROC curves. To evaluate the contribution of the diabetes component of HSI, the index was recalculated after removing the +2 diabetes adjustment, and its AUC was compared with that of the complete HSI using the DeLong test.

## Results

### Baseline clinical, anthropometric, and biochemical characteristics

Baseline characteristics are summarized in **Table 1**. Patients with active Cushing’s disease were younger than those with MACS (p=0.001) and controls (p=0.04), and CD in remission patients were younger than MACS (p=0.02); no other pairwise age differences were significant. Disease duration differed across groups (p=0.001), being longest in CD in remission and shortest in active CD; among patients in biochemical remission, the median remission duration was 22 [7–27] months.

**Table 1:** Baseline clinical, anthropometric, and biochemical characteristics across the study groups.

Variable	Active CD (n=13)	CD in Remission (n=12)	MACS (n=30)	Controls (n=10)	p-value
Gender, female/male, n (%)	11(84.6)/2(15.4)	12(100)/0(0)	27(90)/3(10)	8(80)/2(20)	0.45
Age ^a^, (years)	43.6 ± 6.8	45.6 ± 9.3	53.8 ± 8.6	52.7 ± 3.9	0.001
Disease duration^b^, months	6 [1.5-17.5]	37.5 [32.5-61.5]	22 [12–36.2]		0.001
Anthropometric measurements					
*BMI*^b^, *kg/m²*	28.6 [25.3-35.7]	33.1 [29.2-42.3]	31.6 [28.5-39.3]	25.3 [24.2-27.9]	0.001
*Waist circumference*^b^, *cm*	94 [85–107]	105 [88–122.7]	104 [97.7–116.5]	87 [81–95]	0.002
SBP^b^, mmHg	120 [113–127]	124 [120–129]	120 [113–140]	120 [110–130]	0.77
DBP^b^, mmHg	79 [72–85]	75 [70–80]	80 [70–90]	70 [70–76]	0.12
Comorbidities					
*Diabetes mellitus*, *n (%)*	7 (53.8)	5 (41.7)	15 (50)	0 (0)	0.03
*Number of antidiabetic therapies*, *median (range)*	1 (0–4)	0 (0–2)	0.5 (0–4)	0 (0-0)	0.04
*Hypertension*, *n (%)*	3 (23.1)	5 (41.7)	19 (63.3)	0 (0)	0.002
*Number of antihypertensive therapies*, *median (range)*	0 (0–4)	0 (0–3)	1 (0–4)	0 (0-0)	0.006
*Dyslipidemia*, *n (%)*	4 (30.8)	3 (25)	9 (30)	0 (0)	0.26
*Number of lipid-lowering therapies, median (range)*	0 (0–2)	0 (0–1)	0 (0–1)	0 (0-0)	0.31
Metabolic parameters					
*Fasting glucose*^b^, *mg/dL*	115 [94–165]	93 [89–101.5]	106 [91–133]	95.5 [83–99]	0.02
*HOMA-IR*^b^	5.3 [2.2–7.7]	2.8 [1.6–6.6]	3.8 [2.2–5.2]	2.2 [1.6–2.4]	0.66
*HbA1c*^b^, *%*	6.4 [5.6–6.7]	5.5 [5.2–5.7]	6 [5.5–6.5]	5.4 [5.1–5.6]	0.002
*Triglycerides*^b^, *mg/dL*	131 [76–177]	126 [85–184]	157 [105–229]	95.5 [83–203]	0.33
*HDL-C*^b^, *mg/dL*	49 [41–59]	50 [38–55]	49 [44–59]	53 [40–66]	0.83
*LDL-C*^b^, *mg/dL*	111 [92–166]	105 [93.2–125]	118 [92–137]	145 [86–164]	0.7
*ALT*^b^, *U/L*	21 [17–30.5]	18.5 [10–24]	16.5 [13–23]	18 [13–27]	0.33
*AST*^b^, *U/L*	17 [15–20]	16.5 [14–20]	17.5 [13–22]	18 [17–20]	0.8
Clinical indices					
*HSI*^b^	43 [38.9–48.7]	43.2 [41.2–53.9]	42.1 [39.3–50.7]	35.1 [32.5–38.8]	0.001
*VAI*^b^	123.1 [74.3–251.5]	153 [82.2–293]	154.9 [107.3–254.8]	79.3 [49.6–202.4]	0.28
*METS-VF*^b^	6.23 [5.8–6.4]	6.5 [6–6.6]	6.58 [6.3–6.8]	6.24 [5.7–6.5]	0.004

P-values in **Table 1** refer to overall comparisons across the four study groups; correlation analyses reported elsewhere were performed separately within the hypercortisolism cohort (n=55). Comparisons involving the control group are descriptive; controls were drawn from a separate institutional cohort and were matched for sex only, not for age or BMI.

**^a^** Results presented as mean ± standard deviation (SD).

**^b^** Results are presented as median and interquartile range [IQR]. ALT, alanine aminotransferase; AST, aspartate aminotransferase; BMI, body mass index; CD, Cushing’s disease; DBP, diastolic blood pressure; HbA1c, glycated hemoglobin; HDL-C, high-density lipoprotein cholesterol; HOMA-IR, homeostasis model assessment of insulin resistance; HSI, hepatic steatosis index; LDL-C, low-density lipoprotein cholesterol; MACS, mild autonomous cortisol secretion; METS-VF, metabolic score for visceral fat; SBP, systolic blood pressure; VAI, visceral adiposity index.

BMI was higher in MACS and CD in remission than in controls (p=0.001 and p=0.003, respectively), with no difference between active CD and controls (p=0.3). Waist circumference was higher in MACS than controls (104 [97.7–116.5] cm vs. 87 [81–95] cm, p=0.002); no significant difference was observed between the remaining group pairs.

Diabetes mellitus, absent in controls, was present in 53.8% of active CD, 41.7% of CD in remission, and 50% of MACS patients (p=0.03), with significant differences in the number of antidiabetic therapies (p=0.04). Glucose-lowering agents with recognized effects on visceral and ectopic fat were used only within the hypercortisolemic groups: SGLT2 inhibitors in eight patients (across all three groups) and GLP-1 receptor agonists in four, all in MACS; no control participant received antidiabetic therapy. Fasting glucose (p=0.02) and HbA1c (p=0.002) differed across groups, the latter being higher in active CD and MACS than controls (p=0.002 and p=0.01, respectively). Hypertension prevalence was highest in MACS (63.3%) and differed across groups (p=0.002), as did the number of antihypertensive therapies (p=0.006). Dyslipidemia prevalence did not differ (p=0.26).

### Hormonal profile across study groups

Hormonal parameters are presented in **Table 2**. Basal serum cortisol was higher in active CD and MACS compared to CD in remission (p<0.001 and p=0.03, respectively), with no difference between MACS and controls or MACS and active CD (all p>0.05). Median DHEAS levels were lowest in MACS, significantly below active CD and controls (p=0.002 and p=0.03, respectively). Median LNSC and UFC were higher in active CD than in both MACS and CD in remission (all p<0.01), with no difference between MACS and CD in remission.

**Table 2:** Basal and dynamic hormonal parameters in patients across the study groups.

Variable	Active CD (n=13)	CD in Remission (n=12)	MACS (n=30)	Controls (n=10)	p-value
Parameters assessed in all groups					
*Morning serum cortisol*, *µg/dL*	16.3 [15.2–19.1]	8.4 [5.9–12.7]	13.4 [10.1–18.3]	12.1 [9.7–13.2]	<0.001
*Plasma ACTH*, *pg/mL*	94 [53–129]	21 [13–44]	8.5 [6.4–13.5]	37 [19–41]	<0.001
*DHEAS*, *µg/dL*	163.5 [96.5–316]	73.6 [32.6–118]	61.7 [37.5–86]	155 [103–192]	<0.001
*Post–1mg DST cortisol*, *µg/dL*	13.5 [3.5–19]	0.7 [0.6–0.9]	2.3 [2–3.2]	0.7 [0.6–0.9]	<0.001
Parameters assessed only in hypercortisolemic groups					
*LNSC*, *µg/dL*	0.35 [0.3–0.59]	0.07 [0.06–0.1]	0.12 [0.06–0.2]	–	<0.001
*UFC*, *µg/24h*	113.5 [44.9–188.2]	14 [9.9–16.5]	22 [16.2–34.2]	–	<0.001
*Post–2-day DST cortisol*, *µg/dL*	10.3 [3.7–19.3]	2.5 [1.9–3.2]	2.3 [2–3]	–	<0.001

Comparisons involving the control group are descriptive; controls were drawn from a separate institutional cohort and were matched for sex only, not for age or BMI.

Results are presented as median and interquartile range [IQR].

LNSC and UFC values represent the mean of two separate measurements.

Controls did not undergo dynamic cortisol testing. ACTH, adrenocorticotropic hormone; CD, Cushing’s disease; DHEAS, dehydroepiandrosterone sulfate; DST, dexamethasone suppression test; LNSC, late-night salivary cortisol; MACS, mild autonomous cortisol secretion; UFC, urinary free cortisol.

### MRI-derived ectopic fat compartments across study groups

**Figure 2:**
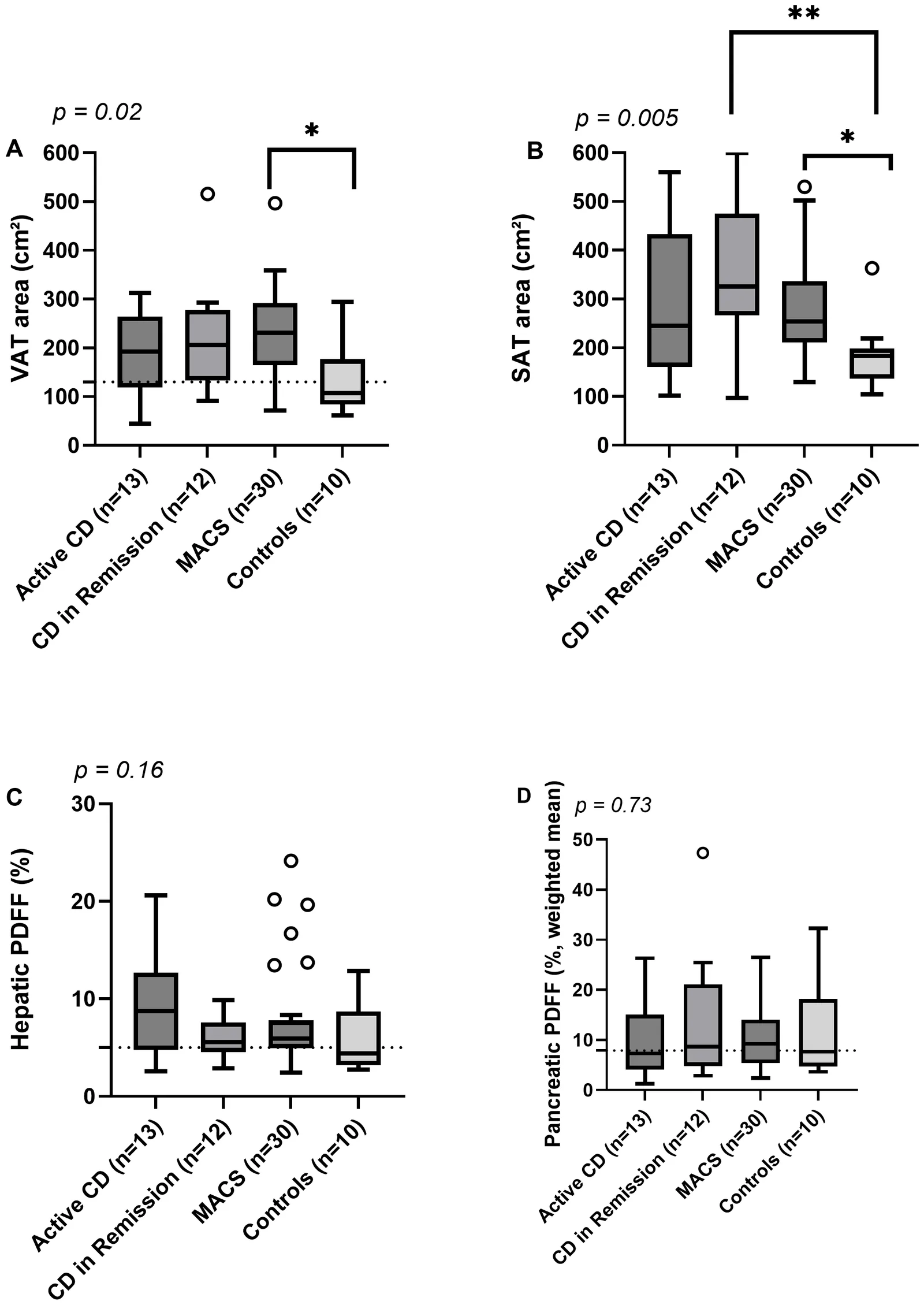
MRI-derived ectopic fat compartments across study groups. Box-and-whisker plots showing **(A)** visceral adipose tissue area, **(B)** subcutaneous adipose tissue area, **(C)** hepatic proton density fat fraction, and **(D)** weighted mean pancreatic proton density fat fraction across active Cushing’s disease (n=13), Cushing’s disease in remission (n=12), mild autonomous cortisol secretion (n=30), and control participants (n=10). Boxes represent median and interquartile range; whiskers extend to 1.5× the interquartile range; outliers are shown as individual data points. Dashed horizontal lines indicate established clinical thresholds: VAT >130 cm² for cardiometabolic risk, hepatic PDFF ≥5% for hepatic steatosis, and pancreatic PDFF ≥7.9% for pancreatic steatosis. *p<0.05, **p<0.01 (pairwise *post-hoc* comparisons with Bonferroni correction).

MRI-derived fat compartment data are summarized in **Table 3** and **Figure 2**. VAT area differed across groups (p=0.02), with the highest median values observed in MACS (230.8 [164.9–292.4] cm²), followed by CD in remission (206.1 [133–277.4] cm²), active CD (192.5 [118.9–264.3] cm²), and controls (107.4 [83.6–176.5] cm²); pairwise comparison revealed a significant difference only between MACS and controls (p=0.01). SAT area also differed across groups (p=0.005), with higher values in CD in remission (325.6 [266.8–475.3] cm²) and MACS (253.7 [211.3–336.8] cm²) compared to controls (182.9 [136.2–198.3] cm²; p=0.002 and p=0.04, respectively), while no significant difference was observed between active CD and controls (p=0.3). Hepatic PDFF and pancreatic PDFF did not differ significantly across groups in any compartment (all p>0.05), although median hepatic PDFF exceeded the 5% steatosis threshold in all three hypercortisolemic groups but not controls (4.4%).

**Table 3:** MRI-derived measures of visceral, subcutaneous, and ectopic fat in patients across the study groups.

Variable	Active CD (n=13)	CD in Remission (n=12)	MACS (n=30)	Controls (n=10)	p-value
Visceral adipose tissue, cm²	192.5 [118.9–264.3]	206.1 [133–277.4]	230.8 [164.9–292.4]	107.4 [83.6–176.5]	0.02
Subcutaneous adipose tissue, cm²	245.4 [160.6–433]	325.6 [266.8–475.3]	253.7 [211.3–336.8]	182.9 [136.2–198.3]	0.005
Hepatic PDFF, %	8.7 [4.7–12.7]	5.5 [4.5–7.5]	5.9 [4.9–7.7]	4.4 [3.1–8.7]	0.16
Pancreatic fat					
*Pancreatic head PDFF*, *%*	5.8 [2–10.3]	11.6 [3.7–19.3]	8.4 [5.4–17.3]	5.2 [4–15.2]	0.3
*Pancreatic body PDFF*, *%*	8.3 [3.6–17.5]	8.2 [6.1–13.5]	10.1 [5.1–15.5]	6.5 [5–17.9]	0.9
*Pancreatic tail PDFF*, *%*	6.9 [4.1–11.5]	7 [3.5–17.3]	8.7 [5.3–13.1]	10.2 [5.9–17.8]	0.7

Comparisons involving the control group are descriptive; controls were drawn from a separate institutional cohort and were matched for sex only, not for age or BMI.

Results are presented as median and interquartile range [IQR]. CD, Cushing’s disease; MACS, mild autonomous cortisol secretion; PDFF, proton density fat fraction.

### Correlations of hormonal, metabolic, and clinical parameters with ectopic fat compartments among participants with hypercortisolism

**Figure 3:**
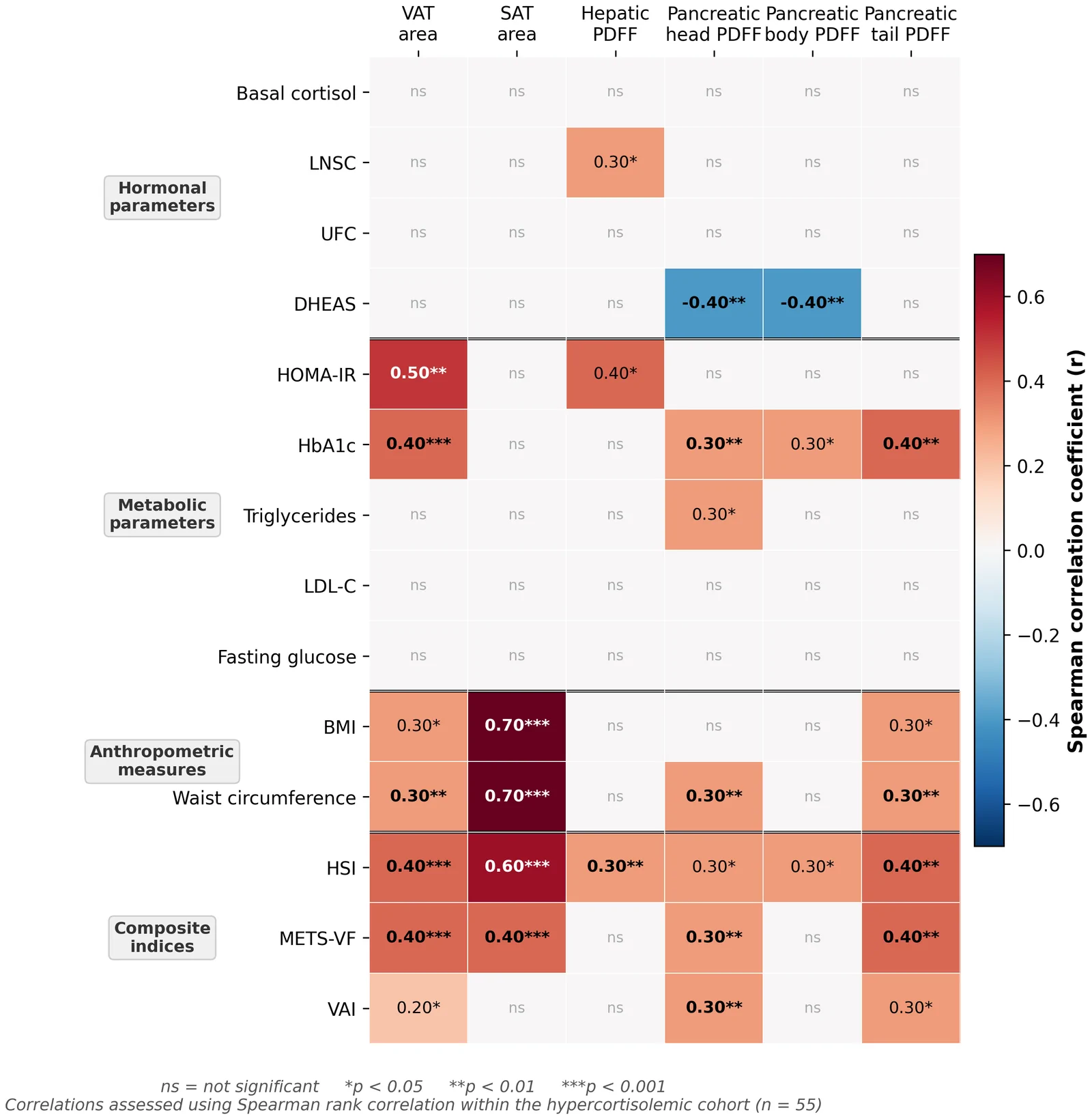
Correlation heatmap of hormonal, metabolic, and clinical parameters with MRI-derived ectopic fat compartments. Spearman rank correlation coefficients between hormonal parameters, metabolic markers, anthropometric measures, and composite clinical indices with visceral adipose tissue area, subcutaneous adipose tissue area, hepatic PDFF, and pancreatic segment PDFF among participants with hypercortisolism (n=55). Cell color intensity reflects the magnitude and direction of the correlation coefficient (red: positive; blue: negative; white: not significant). ns, not significant; *p<0.05; **p<0.01; ***p<0.001. Correlation analyses are exploratory and were not adjusted for multiple comparisons.

Spearman correlations among participants with hypercortisolism (n=55) are shown in **Figure 3**. Among cortisol parameters, only median LNSC correlated with hepatic PDFF (r=0.3, p=0.02); basal cortisol and median UFC showed no association with any compartment. DHEAS correlated inversely with pancreatic head and body PDFF (both r=−0.4, p=0.01).

Among metabolic parameters, within the hypercortisolism cohort (n=55), HOMA-IR was correlated with hepatic PDFF (r=0.4, p=0.02) and VAT area (r=0.5, p=0.009), but not with pancreatic PDFF or SAT (all p>0.05). HbA1c was correlated with VAT area (r=0.4, p=0.001) and with pancreatic PDFF across all three segments (r=0.3–0.4, all p ≤ 0.02), but not with hepatic PDFF or SAT area (all p>0.05).

BMI correlated with VAT area (r=0.3, p=0.02), SAT area (r=0.7, p<0.001), and pancreatic tail PDFF (r=0.3, p=0.02). Waist circumference correlated with VAT area (r=0.3, p=0.01), SAT area (r=0.7, p<0.001), pancreatic head PDFF and tail PDFF (both r=0.3, p=0.01), but not with hepatic or pancreatic body PDFF. Both BMI and HSI were significantly correlated with waist circumference (r=0.9, p<0.001 and r=0.8, p<0.001, respectively).

Among composite indices, HSI was correlated with hepatic PDFF (r=0.3, p=0.01), all three pancreatic segments (r=0.3–0.4, all p<0.04), SAT area (r=0.6, p<0.001), and VAT area (r=0.4, p=0.001). METS-VF was correlated with VAT area and SAT area (both r=0.4, p=0.001), and pancreatic head (r=0.3, p=0.01) and pancreatic tail PDFF (r=0.4, p=0.002), but not hepatic PDFF (p=0.72). VAI correlated only with VAT area (r=0.2, p=0.04) and pancreatic head and tail PDFF (r=0.3, p=0.01 and r=0.3, p=0.02, respectively). Neither age (r=0.24, p=0.2) nor BMI (r=0.28, p=0.1) correlated with VAT area within the MACS subgroup, and neither disease nor remission duration correlated with any compartment.

### Group-level distribution and ROC performance of composite indices

Group-level distributions of the composite indices are shown in **Table 1**. HSI differed across groups (p=0.001), being higher in all three hypercortisolemic groups than controls (active CD vs controls p=0.04; CD in remission vs controls p=0.002; MACS vs controls p=0.002) with no difference among them. METS-VF differed across groups (p=0.004), being higher in MACS than active CD and controls (p=0.008 and p=0.05). VAI did not differ across groups (p=0.28) and was not carried forward to ROC analysis.

**Figure 4:**
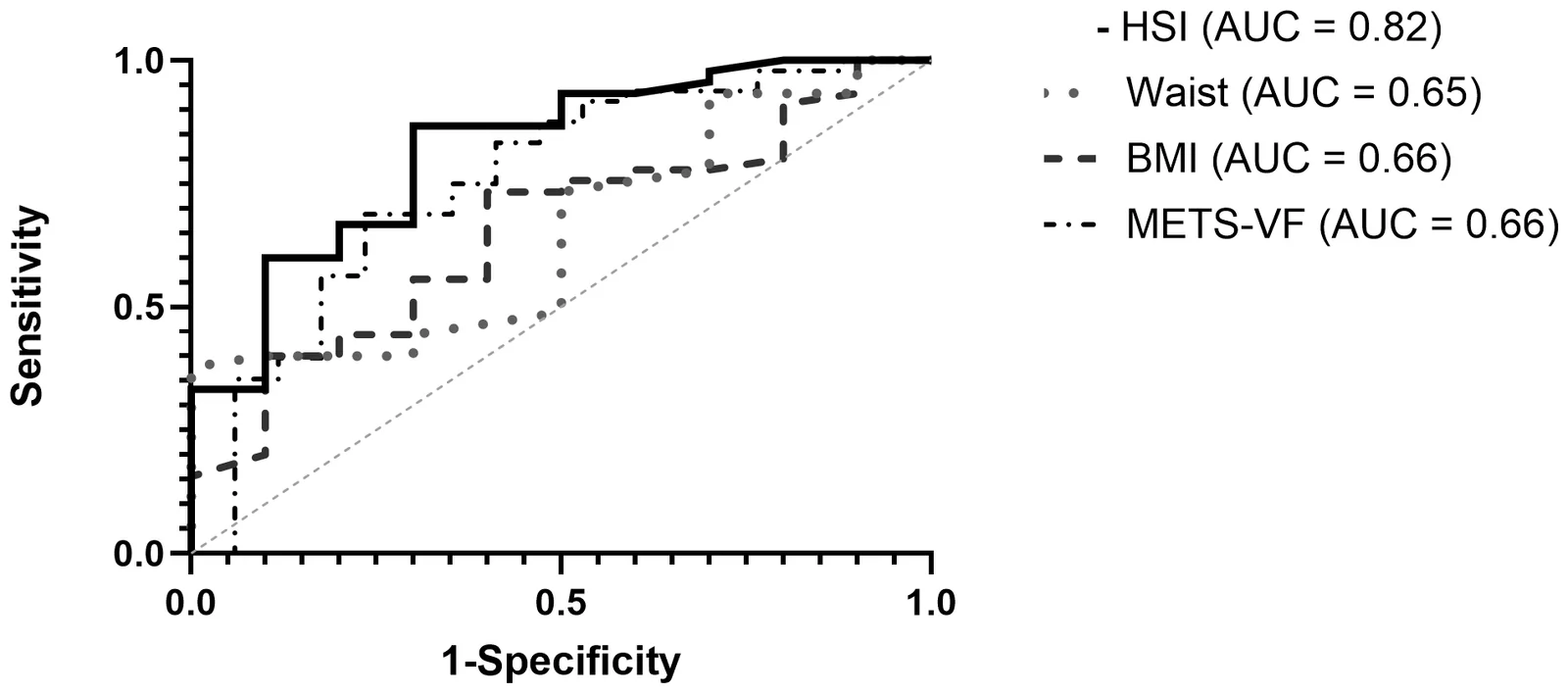
Receiver operating characteristic curves for the identification of MRI-defined visceral adiposity. ROC curves comparing the Hepatic Steatosis Index (HSI), Metabolic Score for Visceral Fat (METS-VF), body mass index (BMI), and waist circumference for the identification of visceral adiposity (VAT >130 cm²) within the hypercortisolemic cohort (n=55). The diagonal dashed line represents the line of no discrimination. AUC, area under the curve. HSI: AUC 0.82 (95% CI: 0.68–0.96); METS-VF: AUC 0.66 (95% CI: 0.4–0.8); BMI: AUC 0.66 (95% CI: 0.5–0.8); waist circumference: AUC 0.65 (95% CI: 0.5–0.8).

ROC analyses were performed among participants with hypercortisolism (n=55) to evaluate the discriminative performance of HSI, METS-VF, BMI, and waist circumference for MRI-defined visceral adiposity (VAT >130 cm²; **Figure 4**). HSI was the only index reaching significance (AUC 0.82, 95% CI: 0.68–0.96, p=0.002). At the cohort-derived optimal cut-off of 39.25, sensitivity was 87% (95% CI: 77–97) and specificity 70% (95% CI: 42–98.4), with a positive predictive value of 93% and negative predictive value of 54% (Youden index 0.6). Internal validation of the HSI analysis showed bootstrap optimism correction (2000 resamples) yielding an optimism-corrected AUC of 0.81 (apparent 0.82;.632 bootstrap AUC 0.82), with the Youden-optimal cut-off stable across all leave-one-out folds and unchanged sensitivity (87%) and specificity (70%). METS-VF (AUC = 0.66, 95% CI: 0.4–0.8, p=0.1), BMI (AUC = 0.66, 95% CI: 0.5–0.8, p=0.1), and waist circumference (AUC = 0.65, 95% CI: 0.5–0.8, p=0.1) did not reach significance. In pairwise DeLong comparisons, the AUC of HSI was significantly higher than that of BMI (z=4.26, p<0.001) and waist circumference (z=2.35, p=0.01), whereas the difference between HSI and METS-VF did not reach statistical significance (p=0.1). To assess the contribution of the diabetes component, HSI was recalculated without the +2 diabetes adjustment. The AUC decreased from 0.82 to 0.78 (ΔAUC 0.04; DeLong p=0.006). A simplified score comprising only the ALT/AST-ratio and BMI components yielded an AUC of 0.79. Among participants with normal waist circumference (n=5), 80% had VAT >130 cm², comparable to the 82% observed in those with elevated waist circumference (n=50; p=1.000).

### Visceral adiposity and HSI performance in participants without obesity

**Figure 5:**
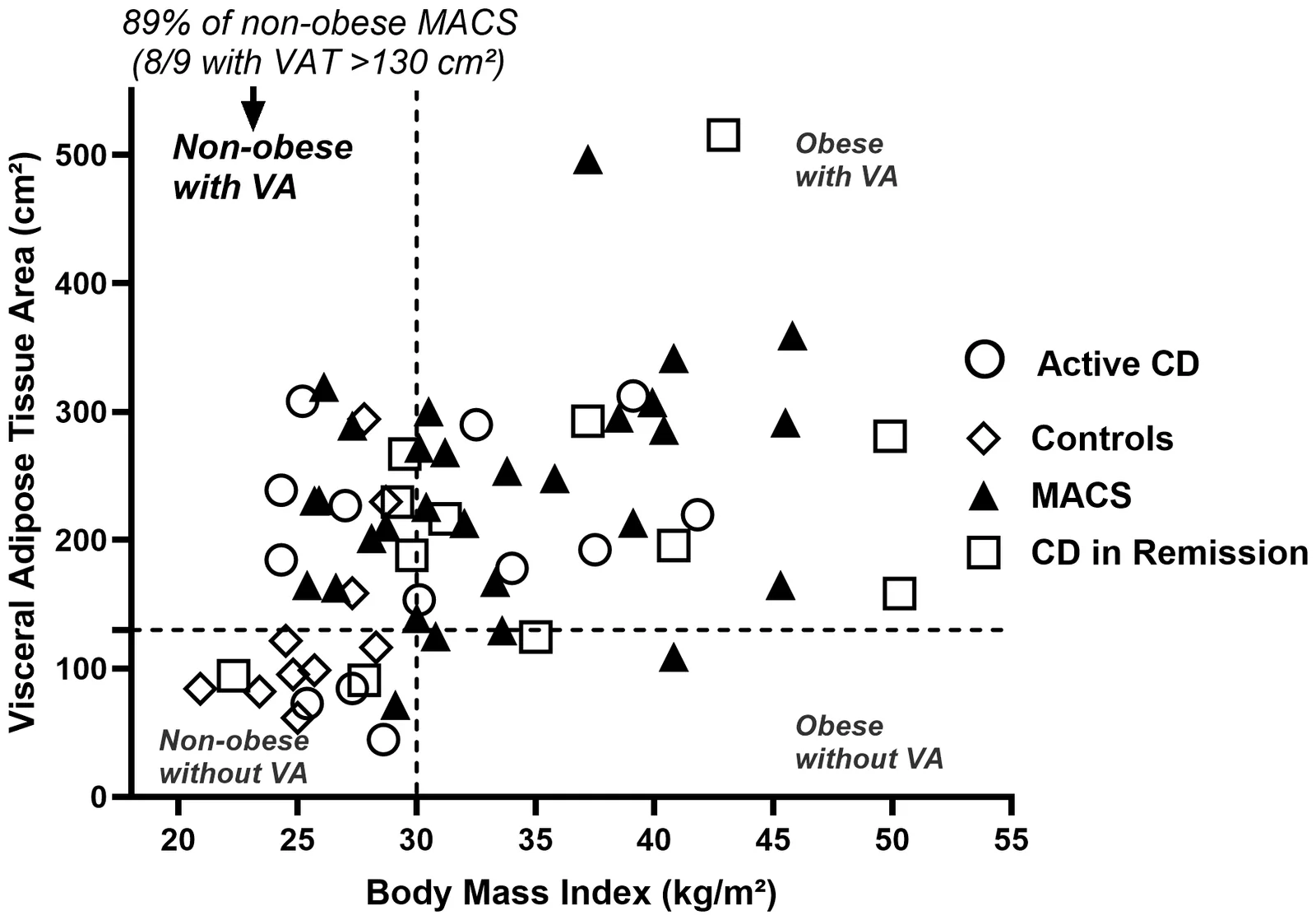
Distribution of body mass index and visceral adipose tissue area across study groups. Scatter plot showing individual BMI and MRI-derived VAT area for each participant. Dashed lines indicate clinical thresholds: BMI = 30 kg/m² (obesity) and VAT = 130 cm² (cardiometabolic risk). The upper-left quadrant identifies non-obese individuals with pathological visceral adiposity. Among participants with MACS without obesity (BMI <30 kg/m²), 89% (8/9) had VAT exceeding the cardiometabolic risk threshold. VA, visceral adiposity.

Among individuals with BMI <30 kg/m² (n=31), visceral adiposity (VAT >130 cm²) was present in 18 patients (58%, **Figure 5**), with the highest prevalence in MACS (89%; 8/9), followed by CD in remission (60%; 3/5), active CD (57.1%; 4/7), and controls (30%; 3/10, p=0.08). When further stratified, in the overweight subgroup (BMI 25–30 kg/m², n=24), visceral adiposity prevalence was highest in MACS (89%; 8/9), followed by CD in remission (75%; 3/4), controls (50%; 3/6), and active CD (40%; 2/5), without significant group differences (p=0.2). In the normal-weight subgroup (BMI <25 kg/m², n=7), the small sample size precluded meaningful inferential statistics. Within the BMI <30 subgroup, HSI risk classification was significantly associated with MRI-defined visceral adiposity (p=0.001): of the 18 participants with VAT >130 cm², HSI correctly identified 10 as high-risk (sensitivity 55.6%), while none of the 13 without visceral adiposity were misclassified (specificity 100%).

### Independent predictors of visceral adiposity: contribution of HSI components

In multiple linear regression with VAT area as the dependent variable and the HSI-constituent variables as covariates (**Table 4**), the model was significant (F(5,49)=5.66, p<0.001, R²=0.36). Diabetes mellitus (B = 83.7, p<0.001) and BMI (B = 4.2, p=0.01) were independent predictors, whereas sex, ALT, and AST were not (all p>0.05).

**Table 4:** Multivariable linear regression analysis of HSI-related variables associated with visceral adiposity.

Variable	B	95% CI	p-value
*Model summary:* R² = 0.36, F(5,49) = 5.66, p <0.001			
Diabetes mellitus	83.7	39−128	<0.001
BMI	4.2	0.93−7.5	0.01
Sex	24.4	−57–106	0.55
ALT	1.2	−1.6–4	0.4
AST	0.7	−5–6	0.8

ALT, alanine aminotransferase; AST, aspartate aminotransferase; BMI, body mass index; CI, confidence interval; HSI, hepatic steatosis index.

## Discussion

In this cross-sectional MRI-based study across the hypercortisolism spectrum, conventional anthropometric measures and dedicated visceral adiposity indices did not demonstrate significant discriminative performance for MRI-defined visceral adiposity in this cohort, whereas HSI, a composite index incorporating liver enzymes and metabolic parameters, performed better. Visceral adiposity was most pronounced in MACS despite lower circulating cortisol than in active Cushing’s disease, and most cortisol-related parameters showed limited association with ectopic fat. Instead, HbA1c and HOMA-IR tracked more consistently with compartment-specific fat deposition, indicating that once hypercortisolism is established, the downstream metabolic alterations were more closely associated with ectopic adiposity than the degree of cortisol excess.

This dissociation between cortisol burden and visceral fat was the central finding. VAT area was highest in MACS and lowest in controls (p=0.01) but did not mirror biochemical hypercortisolism: patients with active Cushing’s disease, despite the highest circulating cortisol, did not show the greatest VAT burden, and those in biochemical remission maintained VAT above the 130 cm² threshold despite normalized cortisol indices. VAT correlated with neither morning serum cortisol, LNSC, UFC, nor disease duration, consistent with a threshold rather than dose-dependent relationship between glucocorticoid excess and visceral adiposity (43). The numerical differences in VAT area across active CD, CD in remission, and MACS may raise the possibility that cumulative cortisol exposure also contributes to visceral adiposity. However, neither disease duration nor, among patients in remission, remission duration was associated with VAT or the other fat compartments in this cohort. Moreover, clinically recorded disease duration may not fully capture true cumulative cortisol exposure, particularly in more indolent conditions such as MACS. Depot-specific activation of 11β-hydroxysteroid dehydrogenase type 1 promotes preferential visceral lipid accumulation whose metabolic consequences operate largely independently of total fat mass (7, 21), consistent with sustained glucocorticoid signaling rather than its magnitude alone (2, 5, 44). Consistent with this, VAT was associated with HOMA-IR (r=0.5, p=0.009) and HbA1c (r=0.4, p=0.001), implicating insulin resistance and glycemic dysregulation as the more proximate correlates. These associations are likely bidirectional and cannot be disentangled in a cross-sectional design, with glucocorticoid excess associated with both visceral adipogenesis and insulin resistance while expanded VAT amplifies insulin resistance through free fatty acid flux and adipokine secretion (45, 46), which may explain the persistence of visceral adiposity in remission. Obesity-associated alterations in cortisol physiology may provide a broader context for these findings. Cortisol can stimulate adipose tissue lipolysis, and glucocorticoid receptor antagonism has been associated with improved adipose insulin sensitivity in insulin-resistant individuals without overt hypercortisolism (47, 48). More recently, Contreras and Falhammar proposed that leptin resistance–related HPA-axis activation may contribute to increased cortisol availability, lipolysis, and ectopic fat deposition in common obesity (49). This may partly overlap metabolically with established endogenous hypercortisolism, although the two conditions are not equivalent. Whether HSI can identify occult visceral adiposity in common obesity or in non-obese individuals with hepatic steatosis remains unknown.

The remaining compartments followed distinct patterns. SAT correlated with BMI and waist circumference (both r=0.7, p<0.001) but with no hormonal or metabolic parameter, indicating that anthropometric measures disproportionately reflect subcutaneous rather than visceral fat; the pronounced SAT in CD in remission most likely reflects persistently elevated BMI. Hepatic PDFF did not differ across groups but exceeded the 5% steatosis threshold (26, 27) in all hypercortisolemic subgroups, correlating selectively with LNSC (r=0.3, p=0.02) and HOMA-IR (r=0.4, p=0.02) but not morning cortisol or UFC; the association with LNSC, a marker of lost circadian nadir, aligns with preclinical evidence that disrupted glucocorticoid rhythmicity drives hepatic lipogenesis (50, 51). Pancreatic fat likewise did not differ across groups, but HbA1c was the only parameter consistently associated with pancreatic PDFF across all three segments (r=0.3–0.4), whereas HOMA-IR showed no association, suggesting pancreatic fat is linked more to chronic glycemic exposure than to systemic insulin resistance, consistent with experimental data on glucotoxic effects on pancreatic lipid homeostasis (52). DHEAS correlated inversely with pancreatic head and body PDFF (r=−0.4). Experimental studies indicate that dehydroepiandrosterone exerts anti-lipogenic and insulin-sensitizing actions via AMPK-dependent pathways, protecting against hepatic steatosis and glycolipid dysregulation (53, 54); an inverse association with pancreatic fat would be directionally consistent with these effects. However, this evidence derives from hepatic models, so its extension to the pancreas remains speculative. Accordingly, this association should be regarded as exploratory and hypothesis-generating, and no mechanistic or causal inference can be made from the present cross-sectional data.

As MRI-PDFF is not routinely available, identifying visceral adiposity must rely on indirect tools. Neither waist circumference nor BMI reached significance in ROC analysis (AUC 0.65 and 0.66), despite modest correlations with VAT, reflecting their far stronger association with subcutaneous fat. The clinical consequence was direct: among patients with normal waist circumference, 80% had VAT above the cardiometabolic threshold, comparable to 82% with elevated values; among participants without obesity, visceral adiposity reached 58% overall and 89% in MACS. These prevalence estimates are threshold-dependent and should be interpreted cautiously, as the 130 cm² VAT cut-off has not been validated specifically in Turkish adults and imaging-derived VAT thresholds vary across populations according to factors including sex, BMI, age, and ethnicity (55). A normal body habitus therefore provides no reassurance against pathological visceral fat in this population. From a clinical perspective, these findings underscore a potential discordance between external body habitus and internal fat distribution, as patients with normal or near-normal anthropometric measures may still harbor substantial MRI-defined visceral adiposity.

Composite indices incorporating biochemical parameters may offer better discrimination. HSI, originally developed for hepatic steatosis screening (19, 56) and previously shown to be elevated across Cushing syndrome subtypes (57, 58), had not been tested for imaging-defined visceral adiposity in hypercortisolism. It was the only index reaching significance in ROC analysis (AUC 0.82, 95% CI: 0.68–0.96), with 87% sensitivity and 93% PPV at a cut-off of 39.25. This threshold is distinct from the original steatosis thresholds and should be regarded as exploratory. Because it was derived from a single cohort, external validation is required before it can be considered for clinical application. Internal validation showed limited estimated optimism in the observed AUC; however, this does not replace validation in an independent cohort, which remains necessary before clinical use. The high PPV suggests that HSI may have potential for identifying individuals at increased likelihood of occult visceral adiposity where MRI is unavailable, whereas the modest NPV (54%) indicates that a low value does not exclude visceral adiposity. Regression identified diabetes mellitus and BMI, both embedded in the HSI formula, as the only independent predictors of VAT. Notably, HSI retained substantial discrimination for visceral adiposity even when its diabetes term was removed (AUC 0.78), suggesting that its performance was not solely attributable to diabetes status. That liver enzymes did not contribute independently suggests HSI’s discriminative value derives from its glycemic and anthropometric components. This utility extended to participants without obesity, in whom HSI classification remained associated with visceral adiposity (p=0.001) with 100% specificity, a property neither BMI nor waist circumference replicated. These findings may also inform a stepwise clinical approach to patients with hypercortisolism and suspected metabolically unfavorable fat distribution. Initial assessment may begin with BMI, waist circumference, and routine metabolic evaluation; however, normal anthropometric findings should not be considered sufficient to exclude visceral adiposity. In patients with persistent metabolic abnormalities or discordance between anthropometric and metabolic findings, composite indices may provide additional information. Among the indices evaluated here, HSI showed significant discrimination and may therefore provide complementary information when advanced imaging is not readily available, although a low HSI does not exclude visceral adiposity. MRI-based assessment allows direct quantification when clinically required. Given the exploratory nature of the HSI cut-off and the absence of external validation, this sequence should be viewed as a clinically oriented framework rather than a validated diagnostic algorithm.

By contrast, the lipid- and proxy-based indices rest on assumptions that glucocorticoid excess undermines. VAI and METS-VF assume that adiposity-related metabolic derangements parallel visceral fat (17, 18, 59), yet in our cohort triglycerides and LDL-C showed no correlation with VAT and categorical dyslipidemia did not differ across groups, decoupling lipid markers from visceral adiposity. VAI showed no group differences and was not carried to ROC analysis; METS-VF, despite significant group-level differences and a VAT correlation comparable to HSI, did not reach individual-level significance (AUC 0.66) and, unlike HSI, showed no association with hepatic fat. Where glucocorticoid excess uncouples metabolic markers from their adipose correlates, indices calibrated to general-population physiology lose discriminative validity.

This study has strengths and limitations. MRI-PDFF enabled simultaneous quantification of visceral, hepatic, and pancreatic fat across the full hypercortisolism spectrum, and to our knowledge this is the first evaluation of anthropometric and composite indices against MRI-PDFF–defined ectopic fat in this setting. The cohort was single-center and modest (n=65, 10 controls), limiting power for smaller differences and subgroup analyses. No *a priori* power calculation was performed, as the sample size was constrained by disease rarity and the predefined recruitment period. Accordingly, the non-significant discrimination observed for METS-VF, BMI, and waist circumference should be interpreted cautiously, as limited statistical power and type II error cannot be excluded. For the primary ROC analysis, a *post-hoc* estimate indicated approximately 99% power to detect the observed HSI AUC of 0.82 against a null value of 0.5 (two-sided α=0.05), suggesting adequate power to detect an effect of this magnitude. All overt cases were pituitary-dependent, so findings may not extend to adrenal Cushing syndrome. The control group was sex-matched (female proportion comparable across groups, p=0.45) but was not matched for age or BMI and, by design, excluded individuals with diabetes and hypertension. As controls were derived from a pre-existing institutional cohort, matching was limited to sex. These baseline differences may confound comparisons involving the control group; therefore, such between-group comparisons should be regarded as descriptive and interpreted cautiously. The principal correlation, ROC, and regression analyses were restricted to the hypercortisolism cohort (n=55) and did not include the control group. Within-group analyses further argued against age or BMI as primary VAT drivers in MACS. The cross-sectional design precludes causal inference, and cumulative cortisol exposure could not be quantified. In addition, MRI and biochemical assessments were not always concurrent (median interval, 28 [14–49] days), which may have introduced temporal variability in the observed associations, although disease status and treatment remained unchanged between assessments. Use of glucose-lowering agents with potential effects on visceral and ectopic fat was limited to the hypercortisolism groups, with GLP-1 receptor agonist use confined to MACS. These therapies may have influenced both metabolic and imaging measures; although their expected effects would generally tend to attenuate adiposity-related abnormalities, the magnitude and direction of this potential confounding cannot be determined in this cross-sectional study. The 130 cm² VAT threshold is not validated in Turkish adults; threshold-dependent results, including the HSI ROC analysis and prevalence estimates, should therefore be interpreted with caution pending local validation, whereas the continuous correlation and regression analyses use VAT area directly and do not depend on this threshold. The cohort was predominantly female (89%), consistent with the known female predominance of Cushing’s disease and MACS but potentially limiting generalizability to men. Ultrasonography, a widely accessible modality particularly for the assessment of hepatic steatosis, was not included in the present imaging protocol; its complementary role alongside MRI-based assessment may warrant dedicated evaluation in future studies. Pancreatic PDFF was derived as a weighted mean from published segment-volume estimates rather than direct volumetric segmentation, which may introduce measurement error and affect steatosis classification. However, segment-specific PDFF values were also evaluated separately, and this limitation does not directly affect the primary HSI–VAT analyses. Future studies may also explore artificial intelligence (AI)-assisted MRI segmentation and quantitative analysis of visceral and ectopic fat compartments, which may facilitate scalability and reduce operator dependence; however, further validation would be needed before such approaches could be considered for routine clinical use. The HSI cut-off remains exploratory. Pairwise DeLong testing showed a significantly higher AUC for HSI than for BMI and waist circumference, whereas the difference between HSI and METS-VF was not statistically significant. The exploratory correlation analyses were not adjusted for multiple comparisons; therefore, particularly weaker associations should be interpreted cautiously as hypothesis-generating findings.

Across the hypercortisolism spectrum, conventional anthropometric measures and dedicated visceral adiposity indices did not reach significance for MRI-defined visceral adiposity, whereas HSI performed better, including among participants without obesity in whom body habitus was least informative. The absence of associations between circulating cortisol and ectopic fat, alongside compartment-specific links with insulin resistance and glycemic burden, suggests that the metabolic sequelae of chronic glucocorticoid exposure, rather than its biochemical intensity, are more closely associated with ectopic fat. Visceral adiposity was present in most patients with normal or near-normal anthropometry, particularly in MACS without obesity, supporting HSI as an accessible complement to anthropometric evaluation for identifying occult visceral adiposity; whether it improves risk stratification warrants prospective evaluation in larger cohorts.

## Data Availability

The raw data supporting the conclusions of this article will be made available by the authors, without undue reservation.
