# Supplementary material for: Clinical indices and anthropometric measures for magnetic resonance imaging-defined visceral adiposity across the hypercortisolism spectrum
Source: Front Endocrinol (Lausanne). 2026 Sep 9;17:1959212. doi: 10.3389/fendo.2026.1959212 (PMC13598908; doi:10.3389/fendo.2026.1959212)
Supplement: Supplementary file 1 [file SupplementaryFile1.docx]

***Supplementary Material***

**1 Supplementary Methods**

**1.1 MRI Acquisition Parameters**

MRI-based proton density fat fraction measurements were obtained using a chemical shift-based mDixon-Quant sequence. Imaging parameters included a 3D breath-hold mDixon-Quant acquisition using a Cartesian Fast Field Echo (FFE) readout with a repetition time (TR) of 5.7 ms, six echo times (TE1 = 0.98 ms, ΔTE = 0.70 ms; 0.98–4.48 ms), a flip angle of 5°, a field of view of 400 × 350 mm, an acquisition matrix of 160 × 142, an acquired voxel size of 2.5 × 2.46 × 6.0 mm and a reconstructed voxel size of 2.08 × 2.08 × 3.0 mm, and a slice thickness of 4 mm with overlapping reconstruction. All acquisitions were performed during breath-hold (scan time 16.9 s) to minimize motion-related artifacts.

**1.2 Pancreatic Segmentation**

The pancreas was assessed in three anatomically defined segments: the head (adjacent to the duodenum), the body (extending from the pancreatic neck to the left of the superior mesenteric vessels and anterior to the aorta), and the tail (the distal portion reaching the splenic hilum).

**1.3 Interobserver Agreement — Bland–Altman Analysis**

Bland–Altman analysis demonstrated minimal bias between readers across all compartments: liver (bias=−0.08, LoA=−1.65 to 1.49), pancreatic head (bias=0.51, LoA=−2.40 to 3.42), pancreatic body (bias=0.32, LoA=−2.43 to 3.08), and pancreatic tail (bias=0.40, LoA=−3.15 to 3.96).

**1.4 Biochemical and Hormonal Assay Methods**

Plasma glucose was measured using the enzymatic hexokinase method (Gluc Hk Gen 3; Roche Diagnostics) on the Cobas 8000 c702 analyzer. HbA1c was determined by turbidimetric inhibition immunoassay (Tina-quant HbA1c Gen 3; Roche Diagnostics) on the same platform. Serum insulin was quantified by electrochemiluminescence immunoassay (ECLIA; Elecsys Insulin; Roche Diagnostics) on the Cobas 8000 e801 analyzer. Serum cortisol, ACTH, and DHEAS were measured by ECLIA (Elecsys Cortisol, Elecsys ACTH, and Elecsys DHEA-S, respectively; Roche Diagnostics) on the e801 platform. Salivary cortisol and urinary free cortisol were quantified using the Elecsys Cortisol II assay (Roche Diagnostics) on the same analyzer. Lipid parameters, including triglycerides, HDL-C, and LDL-C, were assessed using colorimetric and homogeneous enzymatic assays (Trigl, HDLC4, and LDLC3, respectively; Roche Diagnostics) on the c702 system. Serum ALT and AST were measured using kinetic UV assays (ALAT/GPT Gen 2 and ASAT/GOT Gen 2, respectively; Roche Diagnostics) on the c702 analyzer.

**2 Supplementary Figures**

**Supplementary Figure S1.** **Bland–Altman Plots for Interobserver Agreement in MRI-Derived Proton Density Fat Fraction Measurements Across Anatomical Compartments.** Bland–Altman plots demonstrating interobserver agreement for MRI-derived proton density fat fraction (PDFF) measurements across liver, pancreatic head, pancreatic body, and pancreatic tail compartments. Solid lines represent mean bias and dashed lines indicate the 95% limits of agreement (LoA) between the 2 readers.

**Alt text:** Bland–Altman plots demonstrating interobserver agreement for MRI-derived proton density fat fraction measurements across anatomical compartments.
